# The Relationships Between the Eco-Bio-Social Determinants of Dengue Epidemiology in Latin America and the Caribbean: A Scoping Review of the Literature

**DOI:** 10.1007/s10393-025-01764-4

**Published:** 2026-01-30

**Authors:** Aisha Barkhad, Natacha Lecours, Maya Stevens-Uninsky, Lawrence Mbuagbaw

**Affiliations:** 1 Mary Heersink School of Global Health and Social Medicine, McMaster University, 1280 Main St W, Hamilton, ON L8S 4L8 Canada; 2 Global Health Division, International Development Research Centre (IDRC), Ottawa, Canada; 3 Department of Health Research Methods Evidence and Impact, McMaster University, Hamilton, Canada; 4 Department of Anesthesia, McMaster University, Hamilton, ON Canada; 5 Department of Pediatrics, McMaster University, Hamilton, ON Canada; 6 Biostatistics Unit, Father Sean O’Sullivan Research Centre, St Joseph’s Healthcare, Hamilton, ON Canada; 7 Centre for Development of Best Practices in Health (CDBPH), Yaoundé Central Hospital, Yaoundé, Cameroon; 8 Division of Epidemiology and Biostatistics, Department of Global Health, Stellenbosch University, Cape Town, South Africa

**Keywords:** dengue, Latin America, Caribbean, epidemiology, eco-bio-social, scoping review

## Abstract

The factors influencing dengue virus (DENV) transmission by the Aedes aegypti mosquito vector in Latin America and the Caribbean (LAC) are complex and related. The use of a systems thinking, Ecohealth approach for understanding the dynamic linkages between all the ecological, biological, and social (eco-bio-social) factors associated with DENV epidemiology is imperative to better understand the DENV epidemiological system (episystem). The aim of this scoping review was to summarize the evidence for the relationships between the eco-bio-social factors influencing DENV vector dynamics, transmission, and epidemiological outcomes in LAC. The PubMed, SCOPUS, and LILACS databases were searched to collect relevant papers irrespective of design published in English, French, Portuguese, or Spanish. Full-text articles were obtained for the studies that passed the title and abstract screening process. The full-text articles were evaluated to determine if they met the eligibility criteria. Data were extracted using NVivo™ 12, and themes were compiled and communicated narratively. We included 68 studies from 13 different countries from LAC from the published literature from LAC between 2007 and 2022. Studies were classified as eco-biological (n = 24), bio-social (n = 21), eco-social (n = 14), or eco-bio-social studies (n = 9). Ten determinants emerged from the literature and were described, including: temperature; precipitation and humidity; artificial environment; vegetation and land cover; elevation and altitude; human population mobility and human activity; human population density and population demographics; chemical protective measures; seasonality and water storing practices; and urbanization and land use changes. We summarized the evidence on the eco-bio-social determinants of dengue transmission in LAC, with implications for comparable contexts in Asia and Africa. Key gaps remain regarding drivers of transmission and outcomes. Future research may benefit from using transdisciplinary research approaches to investigating *Aedes*-transmissible arboviruses.

**Graphical abstract:**

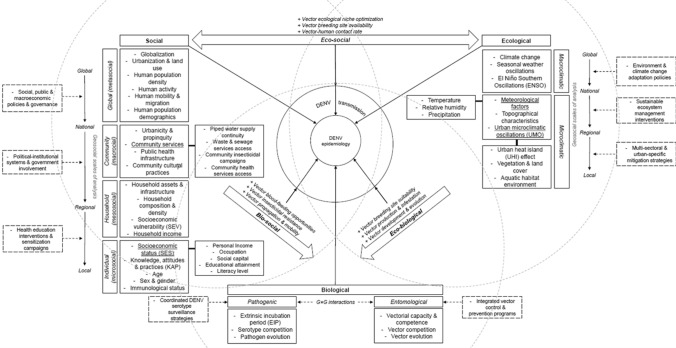

**Supplementary Information:**

The online version contains supplementary material available at 10.1007/s10393-025-01764-4.

## Introduction

Dengue virus (DENV) is a rapidly spreading mosquito-borne, arboviral infectious disease and is transmitted within over 100 countries or territories around the world (Kyle & Harris, 2008; WHO, 2012). Globally, it is estimated that 50–100 million dengue infections occur each year (Guzman et al., 2010). As much as 60% of these reports are from the Americas (Ladner et al., 2017), where the disease has re-emerged over the last 50 years, owing in large part to the re-infestation of the *Aedes aegypti* vector that transmits DENV (Zambrano & San Martin, 2014). This was the consequence of patterns of population growth, migration, a lack of political will, and insufficient finances to sustain an existing *Ae. aegypti* eradication program (Tapia-Conyer et al., 2012). While *Ae. albopictus* is also a competent vector for DENV and has been responsible for several smaller outbreaks (Lambrechts et al., 2010), the majority of DENV serotypes are transmitted by *Ae. aegypti*, (Pereira dos Santos et al., 2017), particularly in Latin America and the Caribbean (LAC).

In LAC, where DENV infection is endemic, documented cases have increased over the decades, making DENV infection one of the region’s most critical threats to public health (Tapia-Conyer et al., 2012). Evidence suggests that the increased DENV occurrence within the region is compounded by a changing climate, as the warming of the globe is enabling the *Ae. aegypti* vector to not only proliferate at escalated rates (Jones et al., 2008), but also exceed their current geographical bounds to reach new, immunologically vulnerable populations (Iwamura et al., 2020). Research demonstrating the bioecological link between DENV vector life cycles and environmental factors are on the rise (Bowman et al., 2014; Morin et al., 2013). These studies have demonstrated that vectors are intrinsically dependent on temperature for their life cycle, reproducibility, and feeding behavior, which regulate their potential to transmit DENV. In addition, there exist anthroposocial drivers of DENV transmission including changes to health infrastructure and policies (Haider & Turner, 2015, and, distinctly, changes to the movement of populations into more dense, urban areas (Alirol et al., 2011). The process of urbanization is generating inequalities by shaping cities defined by poverty, overcrowding, inadequate housing, and lack of basic services such as water, sanitation, and hygiene (PAHO, 1997), all the while seamlessly forming superlative breeding sites for DENV vectors (Gubler, 2004). Some evidence also suggests that, through systems of land use changes, human–environment interactions are shifting, which may have consequences for vector–human contact rates and vector species composition (Johansen et al., 2021).

As dengue represents a vast public health and socioeconomic challenge in LAC, a multisectoral and integrated response is required at all levels. However, a significant challenge stems from the intricacy and heterogeneity of DENV epidemiological systems (episystems; Tabachnick, 2010). Of primary importance will be the use of a systems thinking, Ecohealth approach for understanding the dynamic linkages between all the ecological, biological, and social (eco-bio-social) factors associated with DENV epidemiology. The interdependence of these factors underscores the need for identifying the relationships between the eco-bio-social determinants of dengue in the LAC region. Explicitly integrating eco-bio-social determinants, the Ecohealth approach provides a more comprehensive understanding of dengue transmission that can inform context-specific policies and practices such as targeted vector control, urban planning, and community engagement, leading to more sustainable and effective prevention and control programs and public health policies.Therefore, the aim of this scoping review is to summarize the published literature on the relationships between the ecological, biological, and social determinants of DENV vector dynamics, transmission, and epidemiological outcomes in LAC.

## Methodology

This study extends our earlier scoping review which classified the separate ecological, biological, and social determinants of dengue transmission and epidemiological outcomes in LAC (Barkhad et al., 2025b), providing the foundation for the present methodology and thematic categorization. This scoping work informed our subsequent e-Delphi study conducted with dengue researchers and practitioners of diverse disciplines in LAC to foster a transdisciplinary eco-bio-social conceptual framework for the urban dengue virus episteme (Barkhad et al., 2025a).

## Registration

This review was registered in the Open Science Framework (OSF) Registries (10.17605/OSF.IO/9Z268).

## Eligibility Criteria

We sought published research articles after the release of the Intergovernmental Panel on Climate Change (IPCC) 4th Assessment Report from 2007 to 2022, inclusively (IPCC, 2007). Studies were included if they reported on ecological, biological, and/or social determinants of *Aedes aegypti* vector dynamics, dengue transmission, or dengue-related epidemiological outcomes in 33 countries and 15 dependencies in LAC. We excluded studies that focused exclusively on clinical management or laboratory virology without reference to eco-bio-social determinants, and studies where dengue was not a primary outcome. We included qualitative, quantitative, and mixed method studies, short communications of findings, and discussion papers from LAC, in English, French, Portuguese or Spanish. We excluded opinion pieces, books and book chapters, systematic reviews, meta-analyses, pre-print articles and manuscripts and gray literature (Table S1).

## Data Sources and Search Strategy

In September 2022, we searched PubMed, SCOPUS, and Latin American & Caribbean Health Sciences Literature (LILACS). We developed the literature search strategies using medical subject headings (MeSH) and relevant keywords adapted to each of the database (see Supplemental Material for search terms). Other articles were retrieved from bibliographic references of included articles (Table S2, S3, S4).

## Data Management and Selection

Two reviewers independently managed and recorded articles retrieved from the searches in Zotero software 5.0.67. Screening of titles, abstracts, and full-texts was conducted in Covidence (Veritas Health Innovation, Melbourne, Australia) against the predefined eligibility criteria. Any disagreements were resolved through discussion, and if consensus was not reached, a third reviewer adjudicated. Eligible full-text articles were then transferred to NVivo™ 12 (QSR International, Cambridge, USA) for data extraction and thematic synthesis.

## Data Collection and Analysis

Using our predetermined coding rules, we sorted eco-biological, bio-social, eco-social, and eco-bio-social factors and themes in NVivo™ 12. Specifically, study outcomes were dengue disease transmission, risk, incidence, and/or burden among populations in LAC including intersections of: a) climatological and environmental indicators (e.g., precipitation, humidity, temperature, deforestation, land cover, etc.); b) entomological indicators of *Aedes* genus mosquito vectors (e.g., egg laying time, fecundity, etc.); and c) socioeconomic and demographic indicators (e.g., urbanization, migration, poverty, housing, etc.).

We used a data extraction form in Microsoft Excel 2019 (version 16) to acquire information on the characteristics and context of the article (Table S5).

## Results

### Characteristics of the Included Studies

**Figure. 1:**
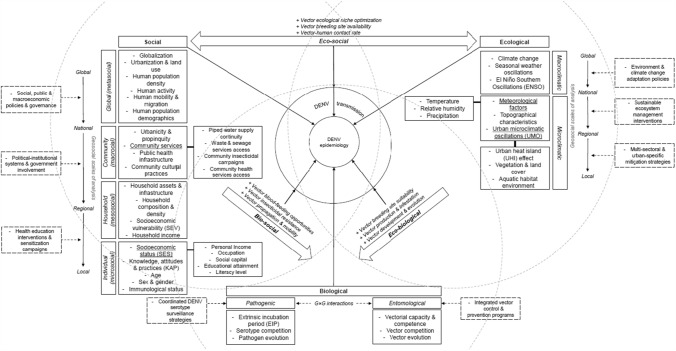
Eco-bio-social factors determining dengue virus transmission and epidemiological outcomes in LAC, based on a scoping review of the literature.

We included 68 articles in this review from 13 different countries, dependencies, and/or territories from LAC (Table 1; Table 2). Most studies were from Brazil (*n* = 25), Colombia (*n* = 10), Mexico (*n* = 6), and Ecuador (*n* = 5). Some studies were conducted in multiple countries (*n* = 3). No studies were published in 2007 and 2010, whereas the most studies were published in 2021 (*n* = 24). The included studies were classified as: eco-biological, bio-social, eco-social, or eco-bio-social studies. Most studies were eco-biological studies (*n* = 24), followed by bio-social studies (*n* = 21), eco-social studies (*n* = 14), and eco-bio-social studies (*n* = 9). Ten main determinants influencing DENV vector dynamics, transmission, and epidemiological outcomes in LAC were identified and are illustrated in Figure. 1.

**Table 1:** Descriptive statistics of the included studies.

Description	Number of studies (n, %)
*Region*	
South America	50 (73.5)
Central America	10 (14.7)
Caribbean Islands	5 (7.4)
Multi-country	3 (4.4)
*Country*	
Brazil	25 (36.8)
Colombia	10 (14.7)
Mexico	6 (8.8)
Ecuador	5 (7.4)
Argentina	3 (4.4)
Venezuela	3 (4.4)
Peru	3 (4.4)
Puerto Rico	3 (4.4)
Multi-country	3 (4.4)
Costa Rica	2 (2.9)
Panama	2 (2.9)
Bolivia	1 (1.5)
Haiti	1 (1.5)
Trinidad and Tobago	1 (1.5)
*Year*	
2007	0 (0.0)
2008	3 (4.4)
2009	5 (7.4)
2010	0 (0.0)
2011	4 (5.9)
2012	3 (4.4)
2013	3 (4.4)
2014	6 (8.8)
2015	8 (11.8)
2016	4 (5.9)
2017	5 (7.4)
2018	3 (4.4)
2019	4 (5.9)
2020	2 (2.9)
2021	14 (20.6)
2022	4 (5.9)
*Classification*	
Ecological	24 (35.3)
Biological	21 (30.9)
Social	14 (20.6)
Eco-bio-social	9 (13.2)

**Table 2:** List of studies included in a scoping review of the literature on the eco-bio-social social determinants of dengue vector dynamics, transmission, and epidemiology in Latin America and the Caribbean.

References	Year of publication	Study setting	Study design	Indicator(s)^†^	Outcome(s)	Factors
Barrera et al	2011	Puerto Rico	Epidemiological study, entomological study, temporal analysis	Temperature, rainfall, container type, vector production, water storing practices	DENV cases	All
Colón-González et al	2013	Mexico	Climatological modelling study	Temperature, precipitation	DENV incidence	All
de Azevedo et al	2018	Brazil	Epidemiological study, spatiotemporal analysis study	Surface temperature, social vulnerability	Vector habitat availability	All
Guagliardo et al	2014	Peru	Epidemiological study, entomological study	Presence of containers, container characteristics, human transportation pathways	Vector presence	All
Ha et al	2021	Ecuador	Entomological study, epidemiologic study	Household conditions, container presence, precipitation, water supply, garbage, unemployment, water volume	Vector pupal density	All
Lozano-Fuentes et al	2012	Mexico	Entomological study, epidemiologic study	Elevation, vegetation index, household composition, air conditioning, garbage collection, water supply	Vector presence	All
Martin et al	2021	Ecuador	Epidemiological study	Temperature, household population, sex of HOH, housing conditions, garbage collection, water supply, occupation, preventative behaviors	Vector presence	All
Ordoñez-Sierra et al	2021	Colombia	Epidemiological study	House index, Breteau index, temperature, precipitation, relative humidity, sex, age, education, healthcare, urban/rural, place of residence	DENV incidence	All
Quintero et al	2009	Colombia	Epidemiological study, cross-sectional study	Gender, education, occupation, SES, health insurance status, KAP, household members	Vector presence	All
Baak-Baak et al	2014	Mexico	Entomological study	Urban condition indicators, container characteristics	Vector presence	Biological, Social
Barrera et al	2008	Puerto Rico	Entomological study, spatial analysis	Vector productivity per household	Vector presence	Biological, Social
Barrera et al	2021	Puerto Rico	Entomological study	Household condition, household location	Vector density	Biological, Social
Diaz-Nieto et al	2013	Argentina	Observational study, entomological study	Presence of containers, vector presence, human transportation pathways	Vector dynamics	Biological, Social
Gonçalves da Silva et al	2012	Multi-country	Epidemiological study, spatial analysis	Human transportation pathways	Vector gene flow	Biological, Social
Guagliardo et al	2015	Peru	Observational study, entomological study	Vector presence, human transportation pathways	Vector infestation	Biological, Social
Guagliardo et al	2019	Peru	Entomological study	Geographic area, human transportation pathways	Vector genetic variability	Biological, Social
Lenhart et al	2008	Haiti	Epidemiological study, entomological study, spatial analysis	Presence of insecticide treated materials	Vector presence, DENV seropositivity	Biological, Social
Lenhart et al	2022	Venezuela	Epidemiological, entomological study	Insecticide treated materials, water storage container covers	Vector presence, DENV seropositivity	Biological, Social
Loroño-Pino et al	2013	Mexico	Epidemiological study, case–control study	Presence of insecticide treated materials	Vector abundance, DENV seropositivity	Biological, Social
Maciel-de-Freitas et al	2014	Brazil	Experimental study, entomological study	Exposure to insecticide	Vector insecticide resistance status	Biological, Social
Manrique-Saide et al	2015	Mexico	Epidemiological study	Presence of insecticide treated materials	Vector infestation	Biological, Social
Nunes et al	2014	Brazil	Experimental study, phylogeographic analysis, evolutionary analysis, spatiotemporal analysis	Vector infestation index, number of scheduled flights, population density	DENV transmission	Biological, Social
Padmanabha et al	2012	Colombia	Epidemiological study, simulation modelling study	Vector presence, human density, human movement	DENV infection, DENV transmission	Biological, Social
Padmanabha et al	2015	Colombia	Epidemiological study, spatial analysis			
	Human movement	DENV transmission	Biological, Social			
Quintero et al	2015	Colombia	Epidemiological study, entomological study	Presence of insecticide treated materials, water storage container covers	Vector presence	Biological, Social
Ryan et al	2019	Ecuador	Epidemiological, entomological study	KAP, household condition	DENV risk, Vector presence	Biological, Social
Vanlerberghe et al	2011	Venezuela	Epidemiological study	Presence of insecticide treated materials, SES, water supply, water storage containers	Vector presence	Biological, Social
Vásquez-Trujillo et al	2021	Colombia	Epidemiological study, observational cross-sectional study	Individual-level factors, household income, property condition, water supply, garbage, Internet, number of people per house, number of rooms	Vector infestation	Biological, Social
Wilke da Silva et al	2018	Brazil	Entomological study, geometric morphometric analysis	Level of urbanization	Vector genetics	Biological, Social
Wilke et al	2017	Brazil	Entomological study,	Level of urbanization	Vector genetics	Biological, Social
Andreo et al	2021	Argentina	Ecological study, epidemiological study, time series clustering	Entomological indicators, vegetation indices, humidity, land cover diversity	DENV incidence	Ecological, Biological
Bennett et al	2021a	Panama	Observational study, entomological surveillance study	Vector ecological niche, seasonality, water temperature, pH, rainfall, humidity, temperature	Vector competition	Ecological, Biological
Bennett et al	2021b	Panama	Experimental study, genetic analyses	Temperature, vegetation, rainfall humidity	Vector adaptability	Ecological, Biological
Beserra et al	2009	Brazil	Experimental study	Temperature	Vector lifecycle	Ecological, Biological
Castillo-Quino et al	2018	Bolivia	Entomological study	Altitude	Vector presence	Ecological, Biological
Chitolina et al	2016	Brazil	Observational study	Oviposition, water characteristics; dissolved oxygen	Vector habitat availability	Ecological, Biological
Custódio et al	2019	Brazil	Entomological study, observational study	Temperature, precipitation, relative humidity	Vector presence	Ecological, Biological
Dibo et al	2008	Brazil	Epidemiological study, entomological study	Vector density, rainfall, temperature	DF incidence	Ecological, Biological
Fuller et al	2009	Costa Rica	Climatological modelling study	Sea surface temperatures, vegetation	DF incidence	Ecological, Biological
Garcia‐Sánchez et al	2017	Colombia	Observational study	Water characteristics, water temperature, pH, dissolved oxygen, planktonic algae concentration	Vector habitat availability	Ecological, Biological
Grech et al	2015	Argentina	Experimental study	Temperature	Vector lifecycle	Ecological, Biological
Hemme et al	2009	Trinidad and Tobago	Experimental study	Water chemistry, water temperature,	Vector presence	Ecological, Biological
Hery et al	2021	Multi-country	Epidemiological study	Water characteristics; water temperature, pH, dissolved oxygen, bacteria concentration	Vector habitat availability	Ecological, Biological
Jaimes-Dueñez et al	2015	Colombia	Experimental study, spatiotemporal study, phylogeographic analysis	Exposure to insecticide, temperature, humidity	Vector genetic variability	Ecological, Biological
Leyton Ramos et al	2020	Colombia	Experimental study, geometric morphometry analysis	Altitude	Vector morphology	Ecological, Biological
Marinho et al	2016	Brazil	Experimental study	Temperature	Vector lifecycle	Ecological, Biological
Moreno-Madriñán et al	2014	Mexico	Entomological study, spatial study	Temperature, rainfall, elevation	Vector abundance	Ecological, Biological
Morin et al	2022	Brazil	Ecological study, epidemiologic study	Temperature, air pressure, specific humidity, relative humidity	DF incidence	Ecological, Biological
Overgaard et al	2017	Colombia	Entomological study, observational study, cross-sectional study	Container characteristics, water characteristics, water temperature, TDS, organic matter, urban/rural residence	Vector abundance	Ecological, Biological
Qualls et al	2016	Ecuador	Entomological study	Presence of sugar sources, vegetation, land cover	Vector distribution	Ecological, Biological
Rodrigues et al	2015	Brazil	Entomological study, epidemiologic study	Temperature, rainfall	Vector abundance	Ecological, Biological
Rubio-Palis et al	2011	Venezuela	Epidemiological study	Temperature, precipitation, relative humidity, vector presence	DENV incidence	Ecological, Biological
Sá et al	2019	Brazil	Experimental study	Exposure to insecticide	Vector insecticide resistance status	Ecological, Biological
Steffler et al	2016	Brazil	Experimental study	Geographic area, climate	Vector genetic variability	Ecological, Biological
Araujo et al	2015	Brazil	Ecological study, epidemiologic study	Temperature, vegetation, population density, SES, household conditions	DENV incidence	Ecological, Social
Bavia et al	2020	Brazil	Epidemiological study, spatiotemporal study	Temperature, rainfall, income	DENV incidence	Ecological, Social
Campos et al	2021	Brazil	Ecological study, epidemiological study	Sex, age, precipitation, temperature, humidity, health vulnerability index	DENV incidence rate	Ecological, Social
Carneiro et al	2017	Brazil	Epidemiological study, cross-sectional observational study, temporal trend analysis	Temperature, pollution	DENV incidence	Ecological, Social
Cunha et al	2021	Brazil	Ecological study, epidemiological study	Vegetation, SEV, SES, population density, building density, land cover, elevation, precipitation, humidity, temperature	DENV incidence	Ecological, Social
Kalbus et al	2021	Brazil	Ecological study	Deforestation, income, household population, access to healthcare	DENV cases	Ecological, Social
Kenneson et al	2017	Ecuador	Epidemiological study	HOH indicators, household conditions, air conditioning, sewer connection, water storing practices	Dengue infection	Ecological, Social
Lowe et al	2021	Brazil	Epidemiological study, spatiotemporal modelling study	Precipitation, temperature, water supply, urbanization	DENV risk	Ecological, Social
Pereira da Silva et al	2022	Brazil	Ecological study, epidemiological study	Vegetation indices	DENV cases	Ecological, Social
Quintero et al	2014	Multi-country	Epidemiological study, entomological study	Household conditions, water supply, water storage, KAP, larval survey,	Vector breeding	Ecological, Social
Romeo-Aznar et al	2022	Brazil	Epidemiological study, modelling study	Population density, seasonality	DENV outbreak	Ecological, Social
Teixeira & Cruz	2011	Brazil	Analytical ecological study	Precipitation, vector infestation, social development index, human development index, inequality, income	DENV incidence	Ecological, Social
Troyo et al	2009	Costa Rica	Ecological study, epidemiologic study	Precipitation, temperature, vegetation, urban condition indicators	DENV incidence	Ecological, Social
Vernal et al	2021	Brazil	Ecological study, spatiotemporal modelling study	Temperature, rainfall, surface pressure, altitude, population density, urbanization, population growth, income, number of healthcare units, water supply, garbage disposal, sewer connection	DENV incidence	Ecological, Social

^†^DENV—dengue virus; DF—dengue fever; HOH—household head; KAP—knowledge, attitudes, and practices; SES—socioeconomic status; SEV—socioeconomic vulnerability; TDS—total dissolved solids.

### Eco-Biological Factors (24 studies)

#### The Effect of Meteorological Factors on Vector Biology and Ecology

##### Temperature

Several studies referenced the impact of ambient temperature on vector biology. For example, one study from Brazil found that at 28 °C and 32 °C, a higher percentage of female *Ae. aegypti* mosquitoes fed on mice blood compared to those at 20 °C and 24 °C (Araujo et al., 2015). Higher temperatures decreased the length of *Ae. aegypti* reproducibility and oviposition cycles (i.e., egg laying time). For instance, a temperature of 16 °C resulted in the lowest fecundity, and the largest number of eggs laid was observed at 28 °C in Brazil (Marinho et al., 2016). According to Beserra and colleagues (2009), the lifecycle development of *Ae. aegypti* and temperature are inversely related. As temperatures increased, the lifecycle development of the vector also decreased from 22 days for the development of egg to adult to 12 days in one Colombian study (Ordoñez-Sierra et al., 2021). Increased temperatures of the aqueous environment increased rates of development of the immature-stage mosquito vector and larval density, and improved the probability of survival (Grech et al., 2015). Shorter development periods at the initial stages of the lifecycle may affect the size of adult females, which is suspected to be an important aspect of vectorial capacity and competence (Leyton Ramos et al., 2020). Changes to temperature may determine the vector’s susceptibility to viral infection since temperature modulates heat-shock proteins, revealing new mosquito phenotypes (Hemme et al., 2009).

##### Precipitation and Humidity

Humidity in the air after rainfall in combination with favorable temperatures may trigger egg hatching (Martin et al., 2021). One study demonstrated that the abundance of *Ae. aegypti* was positively correlated with mean rainfall and total rainfall (Rodrigues et al., 2015). Notably, a time lag between initial rainfall events and human DENV case reporting was documented in some studies. For example, in Venezuela, Rubio-Palis and colleagues (2011) discovered a significant positive relationship between the number of DENV cases two months after rainfall and vector abundance. In urban settings with high human population densities, these environments may provide favorable conditions with respect to the mosquito’s capacity to proliferate. For instance, the accumulations of standing rainwater in urban housing structures (i.e., pots), and waste materials (i.e., discarded cans), provide optimal habitats for the immature stages of the vector (de Azevedo et al., 2018).

#### The Effect of the Physical and Chemical Environment on Vector Biology and Ecology

##### Aquatic environment

Factors related to the aqueous environment including water hardness, volume, potential hydrogen, and presence of organic and inorganic matter impact vector indices (Custódio et al., 2019). The presence of total dissolved solids (TDS) in water storage containers was positively associated with *Ae. aegypti* infestation in Colombia (Overgaard et al., 2017). Allochthonous materials can be inputted into container habitats from rainfall and detritus, as well as leaves and other organic windblown materials. Garcia-Sánchez et al. (2017) found that a larger number of phototrophic microalgae (i.e., Cyanobacteria), in the larval habitats was associated with a larger nutritional supply for immature-stage *Ae. aegypti*. This finding was substantiated by evidence from Ecuador, where contaminated water was positively correlated with higher pupae counts (Ha et al., 2021). The dissolved oxygen content in water was related to the immature development of vectors, where some studies found a negative association between the two variables (Overgaard et al., 2017). However, others found no association between dissolved oxygen content and larval presence (Garcia‐Sánchez et al., 2017). Characteristics of the aqueous environment can affect the ovipositional behavior of adult female mosquitoes. For example, *Ae. aegypti* has been observed to oviposit and develop normally in raw sewage with low levels of dissolved oxygen, suggesting a wide tolerance spectrum (Chitolina et al., 2016).

##### Vegetation and Land Cover

Vegetation canopy refers to localized shading and microclimatic effects (e.g., resting sites, higher humidity) that can support mosquito survival. Specifically, vegetation canopy development regulates temperature variations of mosquito habitats by providing microenvironmental conditions for breeding sites (Bennett et al., 2021a). Shaded areas with vegetation result in cooler temperatures that are suitable for adult mosquitoes resting outside. Shade may also regulate the water temperature in containers, increasing the probability of survival of *Ae. aegypti* larvae (Kenneson et al., 2017). Some studies showed that outdoor habitats without direct sunlight were more likely to contain larvae of *Ae. aegypti* and that tree cover was associated with more suitable larval habitats (Hemme et al., 2009; Troyo et al., 2009). In Argentina, the most important predictors of oviposition temporal patterns were vegetation and humidity indices (Andreo et al., 2021). Tree canopies may decrease sub-canopy wind speed and increase humidity near the ground, which may contribute to greater vectorial competence (Fuller et al., 2009). Moreover, vegetation affords plant sugar meals for mosquitoes, which are a fundamental source of energy for gravid mosquitoes (Qualls et al., 2016). Some findings have shown that lower vegetation cover contributes to the urban heat island (UHI) effect and increases land surface temperatures, rendering the environment more suitable for the vector. For instance, in Brazil, the DENV incidence rate was low in high vegetation cover zones, but high in low vegetation cover zones where the land surface temperature was optimal (Araujo et al., 2015). Other evidence showed that fine-scale genomic variations in *Ae. aegypti* mosquitoes are dependent on vegetation levels. For example, the normalized difference vegetation index and temperature were important predictors of adaptive genomic variation in *Ae. aegypti* with several potential areas of local adaptation in Panama (Bennett et al., 2021b).

##### Elevation and Altitude

Since water temperature is affected by the surrounding ambient temperature, which is higher at lower altitudes, *Ae. aegypti* was historically found in low-elevation urban cities, where studies have confirmed a negative correlation between altitude and DENV incidence (Moreno-Madriñán et al., 2014; Vernal et al., 2021). However, driven by local climate and acclimation capacity, *Ae. aegypti* expanded its elevational range in Bolivia to as high as 2500 m (Castillo-Quino et al., 2018) and as high as 2100 m in Mexico (Lozano-Fuentes et al., 2012). The physical and physiological adaptation of *Ae. aegypti* to a higher altitude is a phenomenon observed in Colombia, where, in the Andean region, Leyton Ramos and colleagues (2020) discovered an altitudinal gradient identifying phenotypic variations of female *Ae. aegypti* wing size and shape.

### Bio-social factors (21 studies)

#### The Effect of Human Population Dynamics on Vector Biology and Behavior

##### Human Population Mobility and Human Activity

The relationships between human social behavior and vector dynamics drive heterogeneity in DENV epidemics. For instance, micro-bursts of DENV infections were driven by movements that led to frequent introductions into heterogeneous urban populations in Colombia (Padmanabha et al., 2015). Human mobility patterns over larger geographical ranges (i.e., migration) contributed significantly to the distant transmission patterns of DENV (Maciel-de-Freitas et al., 2007). In Argentina, the dispersal of *Ae. aegypti* along highways connecting urban centers and popular beaches was cited by Diaz-Nieto et al. (2013) as a likely explanation for the distribution of vectors between densely populated cities, between which were smaller rural towns with abandoned tires that provided vector breeding sites. The immature-stage *Ae. aegypti* can also be transported over long distances in artificial containers due to increased commercial traffic as a result of globalization and trade, where studies have documented immature-stage *Ae. aegypti* vectors on airplanes (Nunes et al., 2014) and on boats (Guagliardo et al., 2015). Steffler et al. (2016) discovered that the movement of people and vehicles between cities promoted the differentiation of *Aedes* populations in Sergipe, Brazil, reflecting an intense gene flow, mediated by the passive dispersion of mosquitoes. Similarly, Guagliardo and colleagues (2019) indicated that *Ae*. *aegypti* gene flow among sub-populations was greatest between locations with heavy boat traffic and lowest between locations with little or no boat or road traffic in Peru. Other population genetic studies further support this evidence in Brazil (Gonçalves da Silva et al., 2012) and in Trinidad and Tobago (Hemme et al., 2009).

##### Human Population Density and Population Demographics

Larger human populations within cities increased the odds of *Ae. aegypti* establishment and habitat availability in the Peruvian Amazon (Guagliardo et al., 2014). Rodrigues et al. (2015) proposed that the number of residents in the household is a factor that attracts female *Ae. aegypti* vectors to the residential environment. These studies demonstrated that more populated environments tend to favor vector proliferation and blood feeding, thus amplifying DENV transmission potential. Further, a modeling study found a significant interaction between human population density, the natural regulatory pattern of *Ae. aegypti*, and DENV transmission dynamics in Colombia (Padmanabha et al., 2012). The model demonstrated that the basic reproductive rate of DENV was likely positively correlated with human density, where variation in secondary infections was largely driven by human-to-mosquito transmission. However, the contrary was discovered in Belo Horizonte, Brazil, where population density demonstrated a negative association with DENV incidence (Cunha et al., 2021). Cunha and colleagues (2021) argued that population density may increase the probability of the community establishment of herd immunity, a human demographic factor important for determining the epidemic potential of DENV. Importantly, Romeo-Aznar et al. (2022) expressed that human density may be a driver of DENV transmission, but it is the variation in population density at fine spatial scales including the number of susceptible and non-susceptible individuals and the interplay with seasonality that is the driving factor.

#### The Effect of Human Preventative Behaviors on Vector Biology and Behavior

##### Chemical Protective Measures.

Community and household chemical vector control interventions including the use of insecticide-treated materials and indoor insecticide spraying, are highly effective measures against mosquito biting (Lenhart et al., 2008, 2022; Manrique-Saide et al., 2015; Vanlerberghe et al., 2011; Quintero et al., 2015). However, local selection pressure has caused a rapid evolution of pyrethroid resistance in *Ae. aegypti* populations across Mexico (Aponte et al., 2013; Solis-Santoyo et al., 2021). Similar findings have been discovered in a population genetic study conducted in Ecuador (Ryan et al., 2019). Sá et al. (2019) discovered a link between resistant vector populations and human population dynamics indicating that the flow of people and cargo may contribute to the dispersion of resistant vectors. Moreover, the use of chemical insecticides is affecting the population genetic composition of *Ae. aegypti*, where one Colombian study discovered that the use of chemical insecticides showed a significant correlation with decreasing genetic diversity in mosquito populations (Jaimes-Dueñez et al., 2015). Few studies have documented the direct relationship between insecticide resistance and DENV incidence. One study conducted in Brazil demonstrated that the intense use of the pyrethroid increased the resistance of the local *Ae. aegypti* population which failed to halt DENV-4 transmission (Maciel-de-Freitas et al., 2014).

### Eco-Social Factors (14 studies)

#### The Effect of Climate and Seasonality on Human Behavior

##### Seasonality and Water Storing Practices

In most areas in LAC, water storage is a societal and cultural practice as a result of the populations’ shared concerns about water scarcity based on historical shortages of water supplies often during periods of prolonged drought. In the dry seasons, buckets, barrels, and containers are the most productive containers for *Ae. aegypti* providing them with the aquatic environments necessary for proliferation, regulating indices of mosquito infestation, the contact rate between humans and vectors, and the potential for a DENV outbreak. For example, Lowe et al. (2021) found that DENV risk following extreme drought was higher in areas that had a higher frequency of water supply shortages in Brazil. This study, among others conducted in Puerto Rico (Barrera, et al., 2011) and in Ecuador (Martin et al., 2021), has shown that whether the role of precipitation is emphasized or diminished in a dry period is likely mediated by human-driven patterns of water storage and use.

#### The Effect of Anthropogenic Activity on Ecosystems

##### Urbanization

Deforestation refers to large-scale loss of vegetation cover and may improve environmental conditions for *Ae. aegypti* dispersal since the loss of vegetation close to urban centers can increase inner-city air temperature and contribute to the UHI effect (Araujo et al., 2015). For example, Pereira da Silva and colleagues (2022) discovered a significant positive trend between DENV fever cases and native loss of vegetation in several Brazilian Cerrado biome states. However, a different report from the Brazilian Amazon rainforest found no significant dose–response association between DENV incidence and deforestation (Kalbus et al., 2021). Moreover, the urbanization process is directly linked to the densification of human populations which has implications for vector dynamics. For example, Wilke et al. (2017) demonstrated that, as urbanized areas expanded and the human population density increased, *Ae. aegypti* populations mimicked this expansion. One other major ecological aspect of the UHI effect is the increase in air pollutants in these regions, which may interfere with the *Ae. aegypti* life cycle development (Carneiro et al., 2017). Urbanization increases the availability of suitable breeding sites for the *Ae. aegypti* vector. For example, Barrera et al. (2008) discovered that *Ae. aegypti* mosquitoes were proliferating in underground septic tanks which may have contributed to island wide DENV endemicity in Puerto Rico. Poor sanitary conditions, including unreliable water supplies and inadequate garbage and waste collection services, characteristic of unplanned urbanization, can favor larval habitat availability and mosquito densities (Fuller et al., 2009). Moreover, *Ae. aegypti* may react to certain selective pressures applied by the urban built environment. One integral study conducted in São Paulo, Brazil by Wilke da Silva and colleagues (2018) demonstrated that environments with higher levels of urbanization exerted stronger selective pressures by imposing the UHI effect, which was further reinforced by social behaviors and cultural practices, that ultimately facilitated microevolutionary changes of the *Ae. aegypti* mosquito’s wing morphology.

## Discussion

### Overview of the Current Evidence

Based on the evidence, a consensus for the regulatory role of the meteorological factors for the transmission of DENV has not yet been reached since the nature of climate and its relationship with dengue outcomes is complex and multi-factorial (Johansson et al., 2009). Universal in the literature, however, was temperature as a significant determinant of DENV vector biting rate, and egg and immature mosquito development, although the effect was not entirely linear or localized. The contrasting evidence regarding the effect of precipitation–humidity and temperature–humidity variables on dengue outcomes suggested that transmission dynamics may be susceptible to environmental fluctuations on a daily or sub-daily scope, likely related to the geophysical and spatial characteristics of the environment. The evidence for topographical dimensions beyond vegetation, land cover, and elevation was absent in this review, yet these factors may represent small-scale geophysical indicators that may help or hinder mosquito dispersal, development, and production (e.g., wind speed). Moreover, the physicochemical properties of the aqueous environment within which vectors oviposit and immature-stage vectors develop demonstrated a unique ecoepidemiological perspective to the DENV episystem, wherein variables such as water temperature, the presence of organic and inorganic matter, and dissolved oxygen, were associated with entomological indicators. However, little was documented about how the presence of conspecifics (i.e., prior oviposition from same species mosquitoes) and congeners (i.e., prior oviposition from same genera of mosquitoes), and other visual, palpable, olfactory, and chemically mediated factors from indicators in the aqueous environment were associated with vector ovipositional behavior (Rey & O’Connell, 2014).

Recognizing the ways in which the urban built environment modified dengue outcomes was a major theme in the literature. This was supported by phylogeographic analyses to link the dynamics of DENV and *Aedes* mosquitoes to their respective geographical and genetic scales. Wilke da Silva and colleagues (2018) demonstrated the sophisticated link between urban landscapes and mosquito morphology, which was mediated by environmental selective pressures. Considered altogether, the relationship between artificial environments and DENV epidemiology is longwinded, and evidence suggested that the link depends on the social characteristics and cultural practices of populations, where humans contribute to urban microclimates that are suitable for the circulation of the DENV transmission chain.

Revealing the bio-anthroposocial mechanisms by which large-scale social factors such as urbanization interact with *Ae. aegypti* dynamics and DENV transmission is of epidemiological importance since social factors govern human behaviors and can change the immunological terrain for urban diseases (Gubler, 2004; Wilke da Silva et al., 2018). The interactions between human mobility and activity and DENV vector establishment within crowded cities merits attention. Human social networks and daily movement can predict clusters of DENV infections since the synanthropic nature of *Ae. aegypti* renders them impressionable to changes to human population dynamics, shifting the DENV episystem flux. Several spatial and spatiotemporal analyses demonstrated how patterns of human movement and migration, population growth and density, and other sociocultural nuances of large-scale impact (i.e., social events) play a potentially large role in the transmission and persistence of DENV. Some evidence suggested that the degree to which human social factors can alter DENV transmission patterns depends on the immunological status of populations and the occurrence of human serotype-specific herd immunity, which may define the epidemic potential of dengue in societies. Moreover, community practices such as the use of chemical insecticides and common spaces sharing were influenced local selection pressures and breeding site availabilities, respectively, altering the biophysiological characteristics of the DENV vector and contributing to their vectorial capacity and competence.

The transmission of DENV may be viewed as a socioecological event that reflects the interactions between the environmental and sociocultural dimensions of the DENV episystem. Catalyzing these interactions are the socioeconomic and demographic characteristics of the population that may position individuals to live, work, or socialize nearby vector habitats. Traditional socioecological research methodologies that aimed to investigate the transmission of DENV used analytic epidemiological and geospatial modeling methods to explore the associations between climate and the social characteristics of human populations. Evidence from the literature demonstrated that seasonal changes to ambient temperatures and patterns of rainfall enhanced vector breeding site availability and influenced seasonal epidemic potential even in the dry seasons, when residents observed water storing practices in some regions. Since DENV vectors exhibit endophilic and endophagic behaviur, and favor anthropophilic habits, the relationship between socioeconomic household conditions and climate may provide useful clues for determining the reach of DENV among human settlements. Moreover, changes to land use as a result of anthropogenic activities such as deforestation may have altered indices of vegetation cover and disrupted natural vector ecosystems while contributing to increased proximate air temperatures near cities. However, research conducted in LAC on the ramifications of human factors on the environment related to this interaction were limited and the scientific evidence to support this conclusion does not exist.

### Limitations

There are some limitations to the present review. First, many articles retrieved from the search strategy were from South America, with significantly less works retrieved from the Caribbean. From those retrieved from South America, most studies were based in Brazil. This may have contributed to selection bias. Second, we did not thoroughly consider the differences between *Ae. aegypti* and *Ae. albopictus* DENV vectors, and largely focused on the urban DENV transmission by *Ae. aegypti*, since this vector is more prevalent in the study region. Thus, we may not have accurately represented the entire eco-bio-social mosaic of DENV transmission since it lacks evidence for the invasive *Ae. albopictus* vector. Finally, we did not consider the specific cultural, economic, political, and social variables defined by differential contexts and historical precedents in particular countries or societies in LAC and at different levels of the human experience.

## Conclusions

We summarized the evidence for the relationships between the eco-bio-social determinants of dengue epidemiology in LAC. The potential that this evidence holds to positively transform the global and public health research landscape for DENV is encouraging. Similarly, this work may be applicable for recognizing other comparable DENV transmission contexts, such as in urban centers of Asia and Africa, where appropriate. However, several knowledge gaps regarding the small and large-scale factors driving DENV transmission and outcomes were made salient. Prospective research in this field may benefit from conducting qualitative research studies that investigate the individual experiences and factors involved in DENV transmission; explore the urban and sylvatic DENV transmission cycle in LAC to make predictions about the impact of deforestation and changes to land use on DENV emergence; and probe the eco-bio-social determinants of other *Aedes*-transmissible arboviruses which co-circulate in LAC (i.e., Chikungunya virus, Zika virus).

## Supplementary Information

Below is the link to the electronic supplementary material.

Supplementary file1 (DOCX 20 KB)

Supplementary file2 (DOCX 48 KB)

## References

[CR1] Alirol E, Getaz L, Stoll B, Chappuis F, Loutan L (2011) Urbanisation and infectious diseases in a globalised world. *The Lancet Infectious Diseases* 11(2):131–141 21272793 10.1016/S1473-3099(10)70223-1 PMC7106397

[CR2] Andreo V, Porcasi X, Guzman C, Lopez L, Scavuzzo CM (2021) Spatial distribution of Aedes aegypti oviposition temporal patterns and their relationship with environment and dengue incidence. *InSects* 12(10):919 34680688 10.3390/insects12100919 PMC8537924

[CR3] Aponte HA, Penilla RP, Dzul-Manzanilla F, Che-Mendoza A, López AD, Solis F, Manrique-Saide P, Ranson H, Lenhart A, McCall PJ, Rodríguez AD (2013) The pyrethroid resistance status and mechanisms in Aedes aegypti from the Guerrero state. *Mexico. Pesticide Biochemistry and Physiology* 107(2):226–234

[CR4] Araujo RV, Albertini MR, Costa-da-Silva AL, Suesdek L, Franceschi NCS, Bastos NM, Katz G, Cardoso VA, Castro BC, Capurro ML, Allegro VLAC (2015) São Paulo urban heat islands have a higher incidence of dengue than other urban areas. *Brazilian Journal of Infectious Diseases* 19:146–155 10.1016/j.bjid.2014.10.004 PMC9425226 25523076

[CR5] de Azevedo, T. S., Bourke, B. P., Piovezan, R., & Sallum, M. A. M. (2018). The influence of urban heat islands and socioeconomic factors on the spatial distribution of Aedes aegypti larval habitats. *Geospatial health, 13*(1). 10.4081/gh.2018.623 29772891

[CR6] Baak-Baak CM, Arana-Guardia R, Cigarroa-Toledo N, Loroño-Pino MA, Reyes-Solis G, Machain-Williams C, Beaty BJ, Eisen L, García-Rejón JE (2014) Vacant lots: productive sites for Aedes (Stegomyia) aegypti (Diptera: Culicidae) in Mérida city. *México. Journal of Medical Entomology* 51(2):475–483 24724299 10.1603/me13209 PMC4064362

[CR7] Barkhad A, Lecours N, Mbuagbaw L (2025a) Developing an eco-bio-social conceptual framework for dengue virus transmission in Latin America and the Caribbean: An e-Delphi study. *PLOS Global Public Health* 5(9):e0004115 40956844 10.1371/journal.pgph.0004115 PMC12440196

[CR8] Barkhad, A., Lecours, N., Stevens-Uninsky, M., & Mbuagbaw, L. (2025b). The Ecological, Biological, and Social Determinants of Dengue Epidemiology in Latin America and the Caribbean: A Scoping Review of the Literature. *EcoHealth*, 1–19. 10.1007/s10393-025-01706-0 PMC12259752 40148718

[CR9] Barrera R, Amador M, Diaz A, Smith J, Munoz-Jordan JL, Rosario Y (2008) Unusual productivity of Aedes aegypti in septic tanks and its implications for dengue control. *Medical and Veterinary Entomology* 22(1):62–69 18380655 10.1111/j.1365-2915.2008.00720.x

[CR10] Barrera R, Amador M, MacKay AJ (2011) Population dynamics of Aedes aegypti and dengue as influenced by weather and human behavior in San Juan. *Puerto Rico. Plos Neglected Tropical Diseases* 5(12):e1378 22206021 10.1371/journal.pntd.0001378 PMC3243685

[CR11] Barrera R, Acevedo V, Amador M (2021) Role of abandoned and vacant houses on Aedes aegypti productivity. *The American Journal of Tropical Medicine and Hygiene* 104(1):145 33021195 10.4269/ajtmh.20-0829 PMC7790113

[CR12] Bavia L, Melanda FN, de Arruda TB, Mosimann ALP, Silveira GF, Aoki MN, Kuczera D, Sarzi ML, Junior WLC, Conchon-Costa I, Pavanelli WR (2020) Epidemiological study on dengue in southern Brazil under the perspective of climate and poverty. *Scientific Reports* 10(1):1–16 32034173 10.1038/s41598-020-58542-1 PMC7005746

[CR13] Bennett KL, McMillan WO, Loaiza JR (2021a) The genomic signal of local environmental adaptation in Aedes aegypti mosquitoes. *Evolutionary Applications* 14(5):1301–1313 34025769 10.1111/eva.13199 PMC8127705

[CR14] Bennett KL, McMillan WO, Enríquez V, Barraza E, Díaz M, Baca B, Whiteman A, Cerro Medina J, Ducasa M, Gómez Martínez C, Almanza A (2021b) The role of heterogenous environmental conditions in shaping the spatiotemporal distribution of competing Aedes mosquitoes in Panama: implications for the landscape of arboviral disease transmission. *Biological Invasions* 23(6):1933–1948 34776763 10.1007/s10530-021-02482-y PMC8550678

[CR15] Beserra EB, Fernandes CR, Silva SADO, Silva LAD, Santos JWD (2009) Efeitos da temperatura no ciclo de vida, exigências térmicas e estimativas do número de gerações anuais de Aedes aegypti (Diptera, Culicidae). *Iheringia. Série Zoologia* 99:142–148

[CR16] Bowman LR, Runge-Ranzinger S, McCall PJ (2014) Assessing the relationship between vector indices and dengue transmission: a systematic review of the evidence. *PLoS Neglected Tropical Diseases* 8(5):e2848 24810901 10.1371/journal.pntd.0002848 PMC4014441

[CR17] Campos NBD, Morais MHF, Ceolin APR, Cunha MDCM, Nicolino RR, Schultes OL, Friche AADL, Caiaffa WT (2021) Twenty-two years of dengue fever (1996–2017): an epidemiological study in a Brazilian city. *International Journal of Environmental Health Research* 31(3):315–324 31468989 10.1080/09603123.2019.1656801

[CR18] Carneiro MAF, Alves BDC, Gehrke FDS, Domingues JN, Sá N, Paixão S, Figueiredo J, Ferreira A, Almeida C, Machi A, Savóia E (2017) Environmental factors can influence dengue reported cases. *Revista Da Associação Médica Brasileira* 63:957–961 29451659 10.1590/1806-9282.63.11.957

[CR19] Castillo-Quino, R., Vallejo-Castro, E., Camacho-Aliaga, A. V., Quiñones-López, A., & Canelas-Urey, H. I. (2018). Adaptation of mosquito Aedes aegypti a 2 550 m s,n.m. Cochabamba, Bolivia. February 2016. *Gaceta Médica Boliviana, 41*(1), 24–30.

[CR20] Chitolina RF, Anjos FA, Lima TS, Castro EA, Costa-Ribeiro MCV (2016) Raw sewage as breeding site to Aedes (Stegomyia) aegypti (Diptera, culicidae). *Acta Tropica* 164:290–296 27640323 10.1016/j.actatropica.2016.07.013

[CR21] Colón-González FJ, Fezzi C, Lake IR, Hunter PR (2013) The effects of weather and climate change on dengue. *PLoS Neglected Tropical Diseases* 7(11):e2503 24244765 10.1371/journal.pntd.0002503 PMC3828158

[CR22] Cunha MDCM, Ju Y, Morais MHF, Dronova I, Ribeiro SP, Bruhn FRP, Lima LL, Sales DM, Schultes OL, Rodriguez DA, Caiaffa WT (2021) Disentangling associations between vegetation greenness and dengue in a Latin American city: Findings and challenges. *Landscape and Urban Planning* 216:104255 10.1016/j.landurbplan.2021.104255 PMC8519391 34675450

[CR23] Custódio, J.M.D.O., Nogueira, L.M.S., Souza, D.A., Fernandes, M.F., Oshiro, E.T., Oliveira, E.F.D., Piranda, E.M. & Oliveira, A.G.D. (2019). Abiotic factors and population dynamic of Aedes aegypti and Aedes albopictus in an endemic area of dengue in Brazil. *Revista do Instituto de Medicina Tropical de São Paulo, 61*. 10.1590/S1678-9946201961018 PMC6453418 30970109

[CR24] Diaz-Nieto LM, Maciá A, Perotti MA, Berón CM (2013) Geographical limits of the southeastern distribution of Aedes aegypti (Diptera, Culicidae) in Argentina. *PLoS Neglected Tropical Diseases* 7(1):e1963 23383351 10.1371/journal.pntd.0001963 PMC3561174

[CR25] Dibo MR, Chierotti AP, Ferrari MS, Mendonça AL, Chiaravalloti Neto F (2008) Study of the relationship between Aedes (Stegomyia) aegypti egg and adult densities, dengue fever and climate in Mirassol, state of São Paulo, Brazil. *Memorias Do Instituto Oswaldo Cruz* 103:554–560 18949325 10.1590/s0074-02762008000600008

[CR26] Fuller DO, Troyo A, Beier JC (2009) El Nino Southern Oscillation and vegetation dynamics as predictors of dengue fever cases in Costa Rica. *Environmental Research Letters* 4(1):014011 10.1088/1748-9326/4/1/014011 PMC2745182 19763186

[CR27] Garcia-Sánchez DC, Pinilla GA, Quintero J (2017) Ecological characterization of Aedes aegypti larval habitats (Diptera: Culicidae) in artificial water containers in Girardot. *Colombia. Journal of Vector Ecology* 42(2):289–297 29125250 10.1111/jvec.12269

[CR28] Gonçalves da Silva A, Cunha ICL, Santos WS, Luz SLB, Ribolla PEM, Abad-Franch F (2012) Gene flow networks among American Aedes aegypti populations. *Evol. Appl.* 5:664–676 23144654 10.1111/j.1752-4571.2012.00244.x PMC3492893

[CR29] Grech MG, Sartor PD, Almirón WR, Ludueña-Almeida FF (2015) Effect of temperature on life history traits during immature development of Aedes aegypti and Culex quinquefasciatus (Diptera: Culicidae) from Córdoba city, Argentina. *Acta Tropica* 146:1–6 25733491 10.1016/j.actatropica.2015.02.010

[CR30] Guagliardo SA, Barboza JL, Morrison AC, Astete H, Vazquez-Prokopec G, Kitron U (2014) Patterns of geographic expansion of Aedes aegypti in the Peruvian Amazon. *PLoS Neglected Tropical Diseases* 8(8):e3033 25101786 10.1371/journal.pntd.0003033 PMC4125293

[CR31] Guagliardo SA, Morrison AC, Barboza JL, Requena E, Astete H, Vazquez-Prokopec G, Kitron U (2015) River boats contribute to the regional spread of the dengue vector Aedes aegypti in the Peruvian Amazon. *PLoS Neglected Tropical Diseases* 9(4):e0003648 25860352 10.1371/journal.pntd.0003648 PMC4393238

[CR32] Guagliardo SAJ, Lee Y, Pierce AA, Wong J, Chu YY, Morrison AC, Astete H, Brosi B, Vazquez-Prokopec G, Scott TW, Kitron U (2019) The genetic structure of Aedes aegypti populations is driven by boat traffic in the Peruvian Amazon. *PLoS Neglected Tropical Diseases* 13(9):e0007552 31532762 10.1371/journal.pntd.0007552 PMC6750575

[CR33] Gubler DJ (2004) Cities spawn epidemic dengue viruses. *Nature Medicine* 10(2):129–130 14760418 10.1038/nm0204-129

[CR34] Guzman MG, Halstead SB, Artsob H, Buchy P, Farrar J, Gubler DJ, Hunsperger E, Kroeger A, Margolis HS, Martínez E, Nathan MB, Pelegrino JL, Simmons C, Yoksan S, Peeling RW (2010) Dengue: a continuing global threat. *Nature Reviews. Microbiology* 8(12 Suppl):S7–S16 21079655 10.1038/nrmicro2460 PMC4333201

[CR35] Ha TA, León TM, Lalangui K, Ponce P, Marshall JM, Cevallos V (2021) Household-level risk factors for Aedes aegypti pupal density in Guayaquil. *Ecuador. Parasites & Vectors* 14(1):1–10 10.1186/s13071-021-04913-0 PMC8425057 34493321

[CR36] Haider M, Turner J (2015) *Variables that May Affect the Transmission of Dengue–A Case Study for Health Management in Asia*. IntechOpen: Topics in Public Health

[CR37] Hemme RR, Tank JL, Chadee DD, Severson DW (2009) Environmental conditions in water storage drums and influences on Aedes aegypti in Trinidad. *West Indies. Acta Tropica* 112(1):59–66 19539592 10.1016/j.actatropica.2009.06.008 PMC4062075

[CR38] Hery L, Guidez A, Durand AA, Delannay C, Normandeau-Guimond J, Reynaud Y, Issaly J, Goindin D, Legrave G, Gustave J, Raffestin S (2021) Natural variation in physicochemical profiles and bacterial communities associated with Aedes aegypti breeding sites and larvae on Guadeloupe and French Guiana. *Microbial Ecology* 81(1):93–109 32621210 10.1007/s00248-020-01544-3 PMC7794107

[CR39] IPCC. (2007). Climate Change 2007: Synthesis Report. Contribution of Working Groups I, II and III to the Fourth Assessment Report of the Intergovernmental Panel on Climate Change [Core Writing Team, Pachauri, R.K and Reisinger, A. (eds.)]. *IPCC, Geneva, Switzerland*, 104 pp.

[CR40] Iwamura T, Guzman-Holst A, Murray KA (2020) Accelerating invasion potential of disease vector Aedes aegypti under climate change. *Nature Communications* 11(1):1–10 10.1038/s41467-020-16010-4 PMC7195482 32358588

[CR41] Jaimes-Dueñez J, Arboleda S, Triana-Chavez O, Gomez-Palacio A (2015) Spatio-temporal distribution of Aedes aegypti (Diptera: Culicidae) mitochondrial lineages in cities with distinct dengue incidence rates suggests complex population dynamics of the dengue vector in Colombia. *PLoS Neglected Tropical Diseases* 9(4):e0003553 25893246 10.1371/journal.pntd.0003553 PMC4403987

[CR42] Johansen IC, Castro MCD, Alves LC, Carmo RLD (2021) Population mobility, demographic, and environmental characteristics of dengue fever epidemics in a major city in Southeastern Brazil, 2007–2015. *Cadernos De Saúde Pública* 37:e00079620 33886707 10.1590/0102-311X00079620

[CR43] Johansson MA, Cummings DA, Glass GE (2009) Multiyear climate variability and dengue—El Nino southern oscillation, weather, and dengue incidence in Puerto Rico, Mexico, and Thailand: a longitudinal data analysis. *PLoS Medicine* 6(11):e1000168 19918363 10.1371/journal.pmed.1000168 PMC2771282

[CR44] Jones KE, Patel NG, Levy MA, Storeygard A, Balk D, Gittleman JL, Daszak P (2008) Global trends in emerging infectious diseases. *Nature* 451(7181):990–993 18288193 10.1038/nature06536 PMC5960580

[CR45] Kalbus A, de Souza Sampaio V, Boenecke J, Reintjes R (2021) Exploring the influence of deforestation on dengue fever incidence in the Brazilian Amazonas state. *PloS One* 16(1):e0242685 33411795 10.1371/journal.pone.0242685 PMC7790412

[CR46] Kenneson A, Beltrán-Ayala E, Borbor-Cordova MJ, Polhemus ME, Ryan SJ, Endy TP, Stewart-Ibarra AM (2017) Social-ecological factors and preventive actions decrease the risk of dengue infection at the household-level: Results from a prospective dengue surveillance study in Machala. *Ecuador. Plos Neglected Tropical Diseases* 11(12):e0006150 29253873 10.1371/journal.pntd.0006150 PMC5771672

[CR47] Kyle JL, Harris E (2008) Global spread and persistence of dengue. *Annual Review of Microbiology* 62(1):71–92 18429680 10.1146/annurev.micro.62.081307.163005

[CR48] Ladner, J., Rodrigues, M., Davis, B., Besson, M. H., Audureau, E., & Saba, J. (2017). Societal impact of dengue outbreaks: stakeholder perceptions and related implications. A qualitative study in Brazil, 2015. *PLoS Neglected Tropical Diseases, 11*(3), e0005366. 10.1371/journal.pntd.0005366 PMC5344327 28278157

[CR49] Lambrechts L, Scott TW, Gubler DJ (2010) Consequences of the expanding global distribution of Aedes albopictus for dengue virus transmission. *PLoS Neglected Tropical Diseases* 4(5):e646 20520794 10.1371/journal.pntd.0000646 PMC2876112

[CR50] Lenhart A, Orelus N, Maskill R, Alexander N, Streit T, McCall PJ (2008) Insecticide-treated bednets to control dengue vectors: preliminary evidence from a controlled trial in Haiti. *Tropical Medicine & International Health* 13(1):56–67 18291003 10.1111/j.1365-3156.2007.01966.x

[CR51] Lenhart A, Castillo CE, Villegas E, Alexander N, Vanlerberghe V, Van Der Stuyft P, McCall PJ (2022) Evaluation of insecticide treated window curtains and water container covers for dengue vector control in a large-scale cluster-randomized trial in Venezuela. *PLoS Neglected Tropical Diseases* 16(3):e0010135 35245284 10.1371/journal.pntd.0010135 PMC8926262

[CR52] Leyton Ramos LM, Aguirre Obando OA, Duque JE, García-Merchán VH (2020) Effect of altitude on wing metric variation of Aedes aegypti (Diptera: Culicidae) in a region of the Colombian Central Andes. *PloS One* 15(8):e0228975 32817690 10.1371/journal.pone.0228975 PMC7440630

[CR53] Loroño-Pino MA, García-Rejón JE, Machain-Williams C, Gomez-Carro S, Nuñez-Ayala G, del Rosario Nájera-Vázquez M, Losoya A, Aguilar L, Saavedra-Rodriguez K, Lozano-Fuentes S, Beaty MK (2013) Towards a Casa Segura: a consumer product study of the effect of insecticide-treated curtains on Aedes aegypti and dengue virus infections in the home. *The American Journal of Tropical Medicine and Hygiene* 89(2):385 23732254 10.4269/ajtmh.12-0772 PMC3741267

[CR54] Lowe R, Lee SA, O’Reilly KM, Brady OJ, Bastos L, Carrasco-Escobar G, de Castro Catão R, Colón-González FJ, Barcellos C, Carvalho MS, Blangiardo M (2021) Combined effects of hydrometeorological hazards and urbanisation on dengue risk in Brazil: a spatiotemporal modelling study. *The Lancet Planetary Health* 5(4):e209–e219 33838736 10.1016/S2542-5196(20)30292-8

[CR55] Lozano-Fuentes S, Hayden MH, Welsh-Rodriguez C, Ochoa-Martinez C, Tapia-Santos B, Kobylinski KC, Uejio CK, Zielinski-Gutierrez E, Delle Monache L, Monaghan AJ, Steinhoff DF (2012) The dengue virus mosquito vector Aedes aegypti at high elevation in Mexico. *The American Journal of Tropical Medicine and Hygiene* 87(5):902 22987656 10.4269/ajtmh.2012.12-0244 PMC3516267

[CR56] Maciel-de-Freitas R, Codeço CT, Lourenço-de-Oliveira R (2007) Body size-associated survival and dispersal rates of Aedes aegypti in Rio de Janeiro. *Medical and Veterinary Entomology* 21(3):284–292 17897370 10.1111/j.1365-2915.2007.00694.x

[CR57] Maciel-de-Freitas R, Avendanho FC, Santos R, Sylvestre G, Araújo SC, Lima JBP, Martins AJ, Coelho GE, Valle D (2014) Undesirable consequences of insecticide resistance following Aedes aegypti control activities due to a dengue outbreak. *PloS One* 9(3):e92424 24676277 10.1371/journal.pone.0092424 PMC3968006

[CR58] Manrique-Saide P, Che-Mendoza A, Barrera-Perez M, Guillermo-May G, Herrera-Bojorquez J, Dzul-Manzanilla F, Gutierrez-Castro C, Lenhart A, Vazquez-Prokopec G, Sommerfeld J, McCall PJ (2015) Use of insecticide-treated house screens to reduce infestations of dengue virus vectors. *Mexico. Emerging Infectious Diseases* 21(2):308 25625483 10.3201/eid2102.140533 PMC4313634

[CR59] Marinho RA, Beserra EB, Bezerra-Gusmão MA, Porto VDS, Olinda RA, Dos Santos CA (2016) Effects of temperature on the life cycle, expansion, and dispersion of Aedes aegypti (Diptera: Culicidae) in three cities in Paraiba. *Brazil. Journal of Vector Ecology* 41(1):1–10 27232118 10.1111/jvec.12187

[CR60] Martin JL, Lippi CA, Stewart-Ibarra AM, Ayala EB, Mordecai EA, Sippy R, Heras FH, Blackburn JK, Ryan SJ (2021) Household and climate factors influence Aedes aegypti presence in the arid city of Huaquillas. *Ecuador. Plos Neglected Tropical Diseases* 15(11):e0009931 34784348 10.1371/journal.pntd.0009931 PMC8651121

[CR61] Moreno-Madriñán MJ, Crosson WL, Eisen L, Estes SM, Estes MG Jr, Hayden M, Hemmings SN, Irwin DE, Lozano-Fuentes S, Monaghan AJ, Quattrochi D (2014) Correlating remote sensing data with the abundance of pupae of the dengue virus mosquito vector, Aedes aegypti, in central Mexico. *ISPRS International Journal of Geo-Information* 3(2):732–749

[CR62] Morin CW, Comrie AC, Ernst K (2013) Climate and dengue transmission: evidence and implications. *Environmental Health Perspectives* 121(11–12):1264–1272 24058050 10.1289/ehp.1306556 PMC3855512

[CR63] Morin CW, Sellers S, Ebi KL (2022) Seasonal variations in dengue virus transmission suitability in the Americas. *Environmental Research Letters* 17(6):064042

[CR64] Nunes MR, Palacios G, Faria NR, Sousa EC Jr, Pantoja JA, Rodrigues SG, Carvalho VL, Medeiros DB, Savji N, Baele G, Suchard MA (2014) Air travel is associated with intracontinental spread of dengue virus serotypes 1–3 in Brazil. *PLoS Neglected Tropical Diseases* 8(4):e2769 24743730 10.1371/journal.pntd.0002769 PMC3990485

[CR65] Ordoñez-Sierra G, Sarmiento-Senior D, Gomez JFJ, Giraldo P, Ramírez AP, Olano VA (2021) Multilevel analysis of social, climatic and entomological factors that influenced dengue occurrence in three municipalities in Colombia. *One Health* 12:100234 33855157 10.1016/j.onehlt.2021.100234 PMC8025047

[CR66] Overgaard HJ, Olano VA, Jaramillo JF, Matiz MI, Sarmiento D, Stenström TA, Alexander N (2017) A cross-sectional survey of Aedes aegypti immature abundance in urban and rural household containers in central Colombia. *Parasites & Vectors* 10(1):1–12 28750651 10.1186/s13071-017-2295-1 PMC5530958

[CR67] Padmanabha H, Durham D, Correa F, Diuk-Wasser M, Galvani A (2012) The Interactive Roles of Aedes aegypti Super-Production and Human Density in Dengue Transmission. *PLoS Neglected Tropical Diseases* 6(8):e1799 22953017 10.1371/journal.pntd.0001799 PMC3429384

[CR68] Padmanabha H, Correa F, Rubio C, Baeza A, Osorio S, Mendez J, Jones JH, Diuk-Wasser MA (2015) Human social behavior and demography drive patterns of fine-scale Dengue transmission in endemic areas of Colombia. *PloS One* 10(12):e0144451 26656072 10.1371/journal.pone.0144451 PMC4684369

[CR69] Pereira da Silva AA, Franquelino AR, Teodoro PE, Montanari R, Faria GA, Ribeiro da Silva CH, Bortoloto da Silva D, Júnior WAR, Muchalak F, Cruz Souza KM, Prudencio da Silva MH (2022) The fewer, the better fare: Can the loss of vegetation in the Cerrado drive the increase in dengue fever cases infection? *PloS One* 17(1):e0262473 35025976 10.1371/journal.pone.0262473 PMC8757950

[CR70] Pereira dos Santos T, Cruz OG, da Silva KAB, de Castro MG, de Brito AF, Maspero RC, de Alcântra R, Dos Santos FB, Honorio NA, Lourenço-de-Oliveira R (2017) Dengue serotype circulation in natural populations of Aedes aegypti. *Acta Tropica* 176:140–143 28743449 10.1016/j.actatropica.2017.07.014

[CR71] Qualls WA, Naranjo DP, Subía MA, Ramon G, Cevallos V, Grijalva I, Gómez E, Arheart KL, Fuller DO, Beier JC (2016) Movement of Aedes aegypti following a sugar meal and its implication in the development of control strategies in Durán. *Ecuador. Journal of Vector Ecology* 41(2):224–231 27860016 10.1111/jvec.12217

[CR72] Quintero J, Carrasquilla G, Suárez R, González C, Olano VA (2009) An ecosystemic approach to evaluating ecological, socioeconomic and group dynamics affecting the prevalence of Aedes aegypti in two Colombian towns. *Cadernos De Saúde Pública* 25:s93–s103 19287871 10.1590/s0102-311x2009001300009

[CR73] Quintero J, Brochero H, Manrique-Saide P, Barrera-Pérez M, Basso C, Romero S, Caprara A, De Lima Cunha JC, Beltrán-Ayala E, Mitchell-Foster K, Kroeger A (2014) Ecological, biological and social dimensions of dengue vector breeding in five urban settings of Latin America: a multi-country study. *BMC Infectious Diseases* 14(1):1–13 24447796 10.1186/1471-2334-14-38 PMC3904013

[CR74] Quintero J, García-Betancourt T, Cortés S, García D, Alcalá L, González-Uribe C, Brochero H, Carrasquilla G (2015) Effectiveness and feasibility of long-lasting insecticide-treated curtains and water container covers for dengue vector control in Colombia: a cluster randomised trial. *Transactions of the Royal Society of Tropical Medicine and Hygiene* 109(2):116–125 25604762 10.1093/trstmh/tru208 PMC4299530

[CR75] Pan American Health Organization (PAHO). (1997). Re-emergence of dengue in the Americas. *Epidemiol Bull. 18*(2). Available from: http://www.paho.org/english/sha/epibul_95-98/be972ree.htm#cau 9376243

[CR76] Rey JR, O’Connell SM (2014) Oviposition by Aedes aegypti and Aedes albopictus: Influence of congeners and of oviposition site characteristics. *Journal of Vector Ecology* 39(1):190–196 24820572 10.1111/j.1948-7134.2014.12086.x

[CR77] Rodrigues MDM, Marques GRAM, Serpa LLN, Arduino MDB, Voltolini JC, Barbosa GL, Andrade VR, de Lima VLC (2015) Density of Aedes aegypti and Aedes albopictus and its association with number of residents and meteorological variables in the home environment of dengue endemic area, São Paulo. *Brazil. Parasites & Vectors* 8(1):1–9 10.1186/s13071-015-0703-y PMC4336725 25890384

[CR78] Romeo-Aznar V, Picinini Freitas L, Gonçalves Cruz O, King AA, Pascual M (2022) Fine-scale heterogeneity in population density predicts wave dynamics in dengue epidemics. *Nature Communications* 13(1):1–9 10.1038/s41467-022-28231-w PMC8864019 35194017

[CR79] Rubio-Palis Y, Pérez-Ybarra LM, Infante-Ruíz M, Comach G, Urdaneta-Márquez L (2011) Influencia de las variables climáticas en la casuística de dengue y la abundancia de Aedes aegypti (Diptera: Culicidae) en Maracay, Venezuela. *Boletin De Malariologia Y Salud Ambiental* 51(2):145–158

[CR80] Ryan, S.J., Lippi, C.A., Nightingale, R., Hamerlinck, G., Borbor-Cordova, M.J., Cruz B, M., Ortega, F., Leon, R., Waggoner, E. & Stewart-Ibarra, A.M. (2019). Socio-ecological factors associated with dengue risk and Aedes aegypti presence in the Galápagos Islands, Ecuador. *International journal of environmental research and public health, 16*(5), p.682. 10.3390/ijerph16050682 PMC6427784 30813558

[CR81] Sá, E.L.R.D., Rodovalho, C.D.M., Sousa, N.P.R.D., Sá, I.L.R.D., Bellinato, D.F., Dias, L.D.S., Silva, L.C.D., Martins, A.J. & Lima, J.B.P. (2019). Evaluation of insecticide resistance in Aedes aegypti populations connected by roads and rivers: the case of Tocantins state in Brazil. *Memórias do Instituto Oswaldo Cruz, 114*. 10.1590/0074-02760180318 PMC6434582 30916115

[CR82] Solis-Santoyo F, Rodriguez AD, Penilla-Navarro RP, Sanchez D, Castillo-Vera A, Lopez-Solis AD, Vazquez-Lopez ED, Lozano S, Black WC IV, Saavedra-Rodriguez K (2021) Insecticide resistance in Aedes aegypti from Tapachula, Mexico: Spatial variation and response to historical insecticide use. *PLoS Neglected Tropical Diseases* 15(9):e0009746 34570792 10.1371/journal.pntd.0009746 PMC8475978

[CR83] Steffler LM, Dolabella SS, Ribolla PEM, Dreyer CS, Araújo ED, Oliveira RG, Martins WFS, La Corte R (2016) Genetic variability and spatial distribution in small geographic scale of Aedes aegypti (Diptera: Culicidae) under different climatic conditions in Northeastern Brazil. *Parasites & Vectors* 9(1):1–9 27716392 10.1186/s13071-016-1814-9 PMC5050563

[CR84] Tabachnick WJ (2010) Challenges in predicting climate and environmental effects on vector-borne disease episystems in a changing world. *Journal of Experimental Biology* 213(6):946–954 20190119 10.1242/jeb.037564

[CR85] Tapia-Conyer R, Betancourt-Cravioto M, Mendez-Galvan J (2012) Dengue: an escalating public health problem in Latin America. *Paediatrics and International Child Health* 32(sup1):14–17 22668444 10.1179/2046904712Z.00000000046 PMC3381443

[CR86] Teixeira TRDA, Cruz OG (2011) Spatial modeling of dengue and socio-environmental indicators in the city of Rio de Janeiro, Brazil. *Cadernos De Saúde Pública* 27:591–602 21519709 10.1590/s0102-311x2011000300019

[CR87] Troyo A, Fuller DO, Calderón-Arguedas O, Solano ME, Beier JC (2009) Urban structure and dengue incidence in Puntarenas, Costa Rica. *Singapore Journal of Tropical Geography* 30(2):265–282 20161131 10.1111/j.1467-9493.2009.00367.x PMC2743112

[CR88] Vanlerberghe V, Villegas E, Oviedo M, Baly A, Lenhart A, McCall PJ, Van der Stuyft P (2011) Evaluation of the effectiveness of insecticide treated materials for household level dengue vector control. *PLoS Neglected Tropical Diseases* 5(3):e994 21468313 10.1371/journal.pntd.0000994 PMC3066153

[CR89] Vásquez-Trujillo, A., Cardona-Arango, D., Segura-Cardona, A. M., Portela-Câmara, D. C., Alves-Honório, N., & Parra-Henao, G. (2021). House-Level Risk Factors for Aedes aegypti Infestation in the Urban Center of Castilla la Nueva, Meta State, Colombia. *Journal of Tropical Medicine*, 2021. 10.1155/2021/8483236 PMC8557085 34725551

[CR90] Vernal, S., Nahas, A. K., Chiaravalloti Neto, F., Prete Junior, C. A., Cortez, A. L., Sabino, E. C., & Luna, E. J. D. A. (2021). Geoclimatic, demographic and socioeconomic characteristics related to dengue outbreaks in Southeastern Brazil: an annual spatial and spatiotemporal risk model over a 12-year period. *Revista do Instituto de Medicina Tropical de São Paulo*, 63. 10.1590/S1678-9946202163070 PMC8494490 34586304

[CR91] Wilk-da-Silva, R., de Souza Leal Diniz, M. M. C., Marrelli, M. T., & Wilke, A. B. B. (2018). Wing morphometric variability in Aedes aegypti (Diptera: Culicidae) from different urban built environments. *Parasites & vectors, 11*(1), 1–9. 10.1186/s13071-018-3154-4 PMC6203966 30367678

[CR92] Wilke ABB, Wilk-da-Silva R, Marrelli MT (2017) Microgeographic population structuring of Aedes aegypti (Diptera: Culicidae). *PloS One* 12(9):e0185150 28931078 10.1371/journal.pone.0185150 PMC5607186

[CR93] World Health Organization (WHO) (2012) *Global strategy for dengue prevention and control, 2012–2020*. Geneva: WHO Press

[CR94] Zambrano B, San Martin JL (2014) Epidemiology of dengue in Latin America. *Journal of the Pediatric Infectious Diseases Society* 3(3):181–182 26625380 10.1093/jpids/piu071

