# Supplementary material for: The Relationships Between the Eco-Bio-Social Determinants of Dengue Epidemiology in Latin America and the Caribbean: A Scoping Review of the Literature
Source: Ecohealth. 2026 Jan 30;23(2):348–66. doi: 10.1007/s10393-025-01764-4 (PMC13287092; doi:10.1007/s10393-025-01764-4)
Supplement: Supplementary file 1 — Supplementary file1 (DOCX 20 KB) [file 10393_2025_1764_MOESM1_ESM.docx]

**Supplemental documentation**

**Table S1.** Criteria for inclusion and exclusion for a scoping review.

| **Description** | **Inclusion** | **Exclusion** |
| --- | --- | --- |
| **Concept** | Studies that:  a) Investigate or describe the relationship between ecological, determinants and dengue disease transmission, risk, incidence, and/or burden among populations in Latin America and the Caribbean including climatological and environmental indicators (e.g., precipitation, humidity, temperature, deforestation, land cover, etc.).  b) Investigate or describe the relationship between biological determinants and dengue disease transmission, risk, incidence, and/or burden among populations in Latin America and the Caribbean including entomological indicators of *Aedes* genus mosquito vectors (e.g., egg laying time, fecundity, etc.).  c) Investigate or describe the relationship between social determinants and dengue disease transmission, risk, incidence, and/or burden among populations in Latin America and the Caribbean including socioeconomic and demographic indicators (e.g., urbanization, migration, poverty, housing, etc.). | Studies that:  a) Describe environment-disease associations without explicitly addressing the impacts of climate change.  b) Report outcome measures different from disease transmission, risk, incidence, and/or burden.  c) Report measures for mosquito vectors other than *Aedes* genus mosquito vectors.  d) Describe arboviral disease epidemiology other than dengue virus. |
| **Type of evidence sources** | Published research articles (qualitative, quantitative, and/or mixed methods), systematic reviews, meta-analyses, short communications of findings, discussion papers. | Grey literature, blogs, social media, correspondence, opinion pieces, books and book chapters, and websites. |
| **Language** | Works published in English, Spanish, or Portuguese languages. | Works published in any language other than English, Spanish, or Portuguese. |
| **Timeframe** | Works published in 2007 or later^a^ | Works published prior to 2007. |
| **Location** | Works from Latin America and the Caribbean^b^ | Works from any location other than Latin America and the Caribbean. |
| **Publication status** | Published works. | Pre-print articles or manuscripts (i.e., prior to peer review). |

^a^ Captures the interval following the release of the IPCC’s monumental 4th Assessment Report.

^b^ There are 33 countries and 15 dependencies in Latin America and the Caribbean today, according to the United Nations (30), and include Anguilla, Antigua and Barbuda, Argentina, Bahamas, Barbados, Belize, Bermuda, Bolivia, Brazil, Cayman Islands, Chile, Colombia, Costa Rica, Cuba, Dominica, Dominican Republic, Ecuador, El Salvador, French Guiana, Grenada, Guadeloupe, Guatemala, Guyana, Haiti, Honduras, Jamaica, Martinique, Mexico, Montserrat, Nicaragua, Panama, Paraguay, Peru, Puerto Rico, Saint Kitts and Nevis, Saint Lucia, Saint Vincent and the Grenadines, Suriname, Trinidad and Tobago, Turks and Caicos Islands, Uruguay, Venezuela, British Virgin Islands, and U.S. Virgin Islands.

**Table S2.** The search strategy applied using the PubMed database on September 20^th^, 2022.

| **Line** | **Search terms** | **Results** |
| --- | --- | --- |
| **1** | ( entomologic* [Title/Abstract]) OR ( egg[Title/Abstract] AND laying [Title/Abstract]) OR ( fecundity [Title/Abstract]) OR ( abundance [Title/Abstract]) OR ( life[Title/Abstract] AND cycle [Title/Abstract]) OR ( feeding[Title/Abstract] AND behaviour [Title/Abstract]) OR ( breed* [Title/Abstract]) OR ( breeding[Title/Abstract] AND site* [Title/Abstract]) OR ( extrinsic[Title/Abstract] AND incubation[Title/Abstract] AND period [Title/Abstract]) OR ( surviv* [Title/Abstract]) OR ( biologic* [Title/Abstract]) | 1118 |
| **2** | ( socioeconomic* [Title/Abstract]) OR ( socio-economic* [Title/Abstract]) OR ( economic* [Title/Abstract]) OR ( sociodemographic* [Title/Abstract]) OR ( socio-demographic* [Title/Abstract]) OR ( demographic* [Title/Abstract]) OR ( urbanization* [Title/Abstract]) OR ( ubanisation* [Title/Abstract]) OR ( migrat* [Title/Abstract]) OR ( poverty [Title/Abstract]) OR ( inequalit* [Title/Abstract]) OR ( social* [Title/Abstract]) |  |
| **3** | (changing[Title/Abstract] AND climate[Title/Abstract]) OR ( climate[Title/Abstract] AND change [Title/Abstract]) OR ( global[Title/Abstract] AND warming [Title/Abstract]) OR variabilit* [Title/Abstract] OR ( meteorologic*[Title/Abstract]) OR ( geograph* [Title/Abstract]) OR ( climatologic* [Title/Abstract]) OR ( environment* [Title/Abstract]) OR (precipitation[Title/Abstract]) OR ( humidity [Title/Abstract]) OR ( temperature [Title/Abstract]) OR ( ecologic*[Title/Abstract]) |  |
| **4** | (transmission [Title/Abstract]) OR ( incidence [Title/Abstract]) OR ( risk* [Title/Abstract]) OR ( burden [Title/Abstract]) OR ( epidemiolog* [Title/Abstract]) |  |
| **5** | (aedes[Title] AND aegypti[Title]) OR ( diptera[Title] AND culicidae [Title]) OR ( dengue [Title]) OR ( dengue[Title] AND virus [Title]) OR (dengue[Title] AND hemorrhagic[Title] and fever [Title]) OR ( denv [Title]) |  |
| **6** | ((Caribbean* OR Anguilla OR (Antigua and Barbuda) OR Argentina OR Bahamas OR Barbados OR Belize OR Bermuda OR Bolivia OR Brazil OR (Cayman AND Islands) OR Chile OR Colombia OR (Costa AND Rica) OR Cuba OR Dominica OR (Dominican AND Republic) OR Ecuador OR (El AND Salvador) OR (French AND Guiana) OR Grenada OR Guadeloupe OR Guatemala OR Guyana OR Haiti OR Honduras OR Jamaica OR Martinique OR Mexico OR Montserrat OR Nicaragua OR Panama OR Paraguay OR Peru OR (Puerto AND Rico) OR (Saint AND Kitts AND Nevis) OR (Saint AND Lucia) OR (Saint AND Vincent AND Grenadines) OR Suriname OR (Trinidad AND Tobago) OR (Turks AND Caicos AND Islands) OR Uruguay OR Venezuela OR (British AND Virgin AND Islands) OR (U.S. AND Virgin AND Islands)) OR (Latin America*)) AND (("2008/01/01"[Date - Publication] : "3000"[Date - Publication])) |  |
| **7** | 1 OR 2 OR 3 |  |
| **8** | 4 AND 7 |  |
| **9** | 5 AND 6 |  |
| **10** | 8 AND 9 |  |

**Table S3.** The search strategy applied using the SCOPUS database on September 20^th^, 2022.

| **Search terms** | **Results** |
| --- | --- |
| ( ( TITLE-ABS-KEY ( ( transmission ) OR ( incidence ) OR ( risk* ) OR ( burden ) OR ( epidemiolog* ) ) ) AND ( ( TITLE-ABS ( ( changing AND climate ) OR ( climate AND change ) OR ( global AND warming ) OR ( climate AND variabilit* ) OR ( meteorologic* ) OR ( geograph* ) OR ( climatologic* ) OR ( environment* ) OR ( precipitation ) OR ( humidity ) OR ( temperature ) OR ( ecologic* ) ) ) OR ( TITLE-ABS ( ( entomologic* ) OR ( egg AND laying ) OR ( fecundity ) OR ( abundance ) OR ( life AND cycle ) OR ( feeding AND behaviour ) OR ( breed* ) OR ( breeding AND site* ) OR ( extrinsic AND incubation AND period ) OR ( surviv* ) OR ( biologic* ) ) ) OR ( TITLE-ABS ( ( socioeconomic* ) OR ( socio-economic* ) OR ( economic* ) OR ( sociodemographic* ) OR ( socio-demographic* ) OR ( demographic* ) OR ( urbanization* ) OR ( ubanisation* ) OR ( migrat* ) OR ( poverty ) OR ( inequalit* ) OR ( social* ) ) ) ) ) AND ( ( TITLE ( ( aedes AND aegypti ) OR ( diptera AND culicidae ) OR ( dengue ) OR ( dengue AND virus ) OR ( dengue AND fever ) OR ( dengue AND hemorrhagic AND fever ) OR ( denv ) ) ) AND ( ( TITLE ( ( caribbean* ) OR ( anguilla ) OR ( antigua AND barbuda ) OR ( argentina ) OR ( bahamas ) OR ( barbados ) OR ( belize ) OR ( bermuda ) OR ( bolivia ) OR ( brazil ) OR ( cayman AND islands ) OR ( chile ) OR ( colombia ) OR ( costa AND rica ) OR ( cuba ) OR ( dominica ) OR ( dominican AND republic ) OR ( ecuador ) OR ( el AND salvador ) OR ( french AND guiana ) OR ( grenada ) OR ( guadeloupe ) OR ( guatemala ) OR ( guyana ) OR ( haiti ) OR ( honduras ) OR ( jamaica ) OR ( martinique ) OR ( mexico ) OR ( montserrat ) OR ( nicaragua ) OR ( panama ) OR ( paraguay ) OR ( peru ) OR ( puerto AND rico ) OR ( saint AND kitts AND nevis ) OR ( saint AND lucia ) OR ( saint AND vincent AND the AND grenadines ) OR ( suriname ) OR ( trinidad AND tobago ) OR ( turks AND caicos AND islands ) OR ( uruguay ) OR ( venezuela ) OR ( british AND virgin AND islands ) OR ( u.s. AND virgin AND islands ) OR ( latin AND america* ) ) PUBYEAR > 2007 ) ) ) | 708 |

**Table S4.** The search strategy applied using the LILACS database on September 22^nd^, 2022.

| **Search terms** | **Results** |
| --- | --- |
| [DENGUE or "dengue virus" or "DENV" and "aedes aegypti"] Palavras do titulo | 181 |
| [ecological or "environmental" or "biological" or "social" or "epidemiological" or "epidemiology"] Palavras do titulo |  |
| [2007 OR "2008" or "2009" or "2010" or "2011" or "2012" or "2013" or "2014" or "2015" or "2016" or "2017" or "2018" or "2019" or "2020" or "2021" or "2022"] Pais, ano de publicacao |  |

**Table S5.** Data extraction table template for a review.

|  | **Item** | **Description** |
| --- | --- | --- |
| **Publication details** |  |  |
|  | Author(s) |  |
|  | Title |  |
|  | WHO Region |  |
|  | Country of origin |  |
|  | Country income level | Low income, lower-middle income, upper-middle income, or high income |
|  | Language(s) of country |  |
|  | Field of study | Epidemiology, entomology, sociology, environmental sciences, public health, etc. |
|  | Publication type | Scientific report, review, commentary, etc. |
|  | Year of data collection |  |
|  | Year of publication |  |
|  | Source of funding | Including role of funders |
| **Study details** |  |  |
|  | Aim of study | Stated rationale, objectives, and purpose |
|  | Study setting | Context and location of focus (e.g., urban, rural) |
|  | Study population | Women, pregnant women, children, elderly, men, etc. |
|  | Type of study | Qualitative, quantitative, mixed methods, etc. |
|  | Study design and methodologies | Cross-sectional study, cohort study, case-control study, etc. |
|  | Sample size | Including sample size calculations and rationale |
|  | Sampling technique | (e.g., convenience or random sampling) |
|  | Variables of interest | Socioeconomic and demographic variables, environmental and climatic variables (e.g., risk factors of interest) |
|  | Types of outcome measures | Measures of association (e.g., odds ratio, relative risks), prevalence, seroprevalence, self-reported outcomes, etc. |
|  | Key findings |  |
|  | Important themes | Themes or dimensions of climate change, dengue virus, and health equity (e.g., One Health approach, ecohealth approach, etc.) |
|  | Study limitations |  |
|  | Conclusions | Future directives and recommendations for policies and programs |
