# Supplementary material for: The Relationships Between the Eco-Bio-Social Determinants of Dengue Epidemiology in Latin America and the Caribbean: A Scoping Review of the Literature
Source: Ecohealth. 2026 Jan 30;23(2):348–66. doi: 10.1007/s10393-025-01764-4 (PMC13287092; doi:10.1007/s10393-025-01764-4)
Supplement: Supplementary file 2 — Supplementary file2 (DOCX 48 KB) [file 10393_2025_1764_MOESM2_ESM.docx]

**Identification of studies via databases and registers**

Records identified from:

PubMed (n = 1118)

SCOPUS (n = 708)

LILACS (n = 181)

Records removed *before screening*:

Duplicate records removed

(n = 493)

**Identification**

Records screened

(n = 1514)

Records excluded (n = 1235)

**Screening**

Reports sought for retrieval

(n = 279)

Reports not retrieved (n = 40)

**Reports excluded** (n = 171)

Wrong disease/vector (n = 29)

Wrong outcomes (n = 61)

Wrong methods (n = 31)

Other (n = 50)

Reports assessed for eligibility

(n = 239)

Studies included in review

(n = 68)

**Included**

*Consider, if feasible to do so, reporting the number of records identified from each database or register searched (rather than the total number across all databases/registers).

**If automation tools were used, indicate how many records were excluded by a human and how many were excluded by automation tools.

*From:*  Page MJ, McKenzie JE, Bossuyt PM, Boutron I, Hoffmann TC, Mulrow CD, et al. The PRISMA 2020 statement: an updated guideline for reporting systematic reviews. BMJ 2021;372:n71. doi: 10.1136/bmj.n71

For more information, visit: <http://www.prisma-statement.org/>
